# Hyperuricaemia, gout and related adverse events associated with antihypertensive drugs: A real-world analysis using the FDA adverse event reporting system

**DOI:** 10.3389/fphar.2022.1045561

**Published:** 2023-01-09

**Authors:** Xue-Feng Jiao, Kunpeng Song, Xueyan Jiao, Hailong Li, Linan Zeng, Kun Zou, Wei Zhang, Huiqing Wang, Lingli Zhang

**Affiliations:** ^1^ Department of Pharmacy, West China Second University Hospital, Sichuan University, Chengdu, China; ^2^ Evidence-Based Pharmacy Center, West China Second University Hospital, Sichuan University, Chengdu, China; ^3^ NMPA Key Laboratory for Technical Research on Drug Products In Vitro and In Vivo Correlation, Chengdu, China; ^4^ Key Laboratory of Birth Defects and Related Diseases of Women and Children, Sichuan University, Ministry of Education, Chengdu, China; ^5^ Department of Cardiovascular Medicine Ward II, Zhengzhou Central Hospital Affiliated to Zhengzhou University, Zhengzhou, China; ^6^ Henan University of Traditional Chinese Medicine, Zhengzhou, China; ^7^ West China Biomedical Big Data Center, West China Hospital, Sichuan University, Chengdu, China; ^8^ Medical Big Data Center, Sichuan University, Chengdu, China; ^9^ Medical Simulation Centre, West China Second University Hospital, Sichuan University, Chengdu, China; ^10^ Chinese Evidence-Based Medicine Center, West China Hospital, Sichuan University, Chengdu, China

**Keywords:** antihypertensive drugs, hyperuricaemia, gout, adverse events, the FDA adverse event reporting system

## Abstract

**Background:** The role of antihypertensive drugs in inducing hyperuricaemia and gout has been a long-term concern in clinical practice. However, clinical studies regarding this issue are limited in number and have yielded inconsistent results. We comprehensively evaluated the association between various antihypertensive drugs and the occurrences of hyperuricaemia, gout and related adverse events (AEs) using the FDA Adverse Event Reporting System (FAERS), aiming to guide the selection of antihypertensive drugs with a goal of minimizing the risk of hyperuricaemia, gout and related AEs.

**Methods:** We used OpenVigil 2.1 to query the FAERS database. Hyperuricaemia, gout and related AEs were defined by 5 Preferred Terms: hyperuricaemia, gout, gouty arthritis, gouty tophus and urate nephropathy. Disproportionality analysis was performed, and a positive signal indicated an association between AEs and antihypertensive drugs.

**Results:** The numbers of antihypertensive drugs with positive signals for hyperuricaemia, gout, gouty arthritis, gouty tophus and urate nephropathy were 46, 66, 27, 8 and 6, respectively. These drugs included diuretics, antihypertensive drugs with central action, α blockers, β blockers, α and β blockers, calcium channel blockers, angiotensin converting enzyme inhibitors, angiotensin II receptor blockers, renin inhibitors, vasodilators, and compound preparations. Furthermore, 42 antihypertensive drugs had positive signal for more than one AEs.

**Conclusion:** Our study suggests that some potassium-sparing diuretics, calcium channel blockers and losartan may be associated with increased risk of hyperuricaemia, gout or related AEs, which is inconsistent with most previous studies. Moreover, Our study also suggests that some antihypertensive drugs with central action, α and β blockers, renin inhibitors and vasodilators may be associated with increased risk of hyperuricaemia, gout or related AEs, which has not been reported in previous studies. These findings complement real-world evidence on the potential risks of hyperuricaemia, gout and related AEs associated with antihypertensive drugs.

## 1 Introduction

Hyperuricaemia and gout are common comorbidities of hypertension. A recent nationwide cross-sectional study in China demonstrated that the prevalence rate of hyperuricaemia in hypertensive patients was up to 38.7% (Liu et al., 2021). Moreover, according to the estimates from the National Health and Nutrition Examination Survey (NHANES), an astonishing 74% of patients with gout have hypertension (Zhu et al., 2012). The presence of hyperuricaemia or gout in hypertensive patients significantly increase the risks of cardiovascular diseases, renal insufficiency and all-cause mortality (Verdecchia et al., 2000; Cicero et al., 2014; Feig, 2014). Therefore, the prevention and treatment of hyperuricaemia and gout in hypertensive patients should arouse enough attention.

The role of antihypertensive drugs in inducing hyperuricaemia and gout has been a long-term concern in clinical practice. However, clinical studies regarding this issue are limited in number and have yielded inconsistent results. For example, for hyperuricaemia, a cross-sectional study in Japan showed that uses of diuretics, β blockers and α blockers were associated with increased risk of hyperuricaemia, while uses of calcium channel blockers, angiotensin converting enzyme inhibitors, angiotensin II receptor blockers, including losartan were not associated with risk of hyperuricaemia (Ueno et al., 2016). However, a nationwide cross-sectional study in China suggested that uses of losartan, valsartan, nifedipine and β blockers were associated with reduced risk of hyperuricaemia, while uses of other antihypertensive drugs including amlodipine and diuretics were not associated with risk of hyperuricaemia (Liu et al., 2021). In addition, for gout, a nested case-control study of data from the UK general practice database showed that diuretics, β blockers, angiotensin converting enzyme inhibitors and non-losartan angiotensin II receptor blockers increased the risk of gout, whereas calcium channel blockers and losartan reduced the risk of gout (Choi et al., 2012). But a population-based cohort study in the United States suggested that non-diuretic antihypertensive drugs significantly reduced the risk of gout (McAdams DeMarco et al., 2012). Thus, the current clinical evidence is insufficient to guide the selection of antihypertensive drugs with a goal of minimizing the risk of hyperuricaemia and gout.

The FDA Adverse Event Reporting System (FAERS) is one of the largest spontaneous reporting databases in the world which contains over twenty million adverse event (AE) reports submitted to FDA, and could comprehensively reflect drug safety profiles in real-world clinical settings (FDA Adverse Event Reporting System (FAERS) Quarterly Data Extract Files, 2022b). Moreover, data mining methods have already been developed for signal detection in the FAERS database, that is, a positive signal indicates a statistical association between a drug and an AE (Duggirala et al., 2016). Furthermore, due to the large sample size, FAERS has enough statistical power to identify rare adverse reactions that can hardly be found in conventional epidemiological studies (Jiao et al., 2020). Thus, in this study, we comprehensively evaluated the association between various antihypertensive drugs and the occurrences of hyperuricaemia, gout and related AEs using the FAERS database, with the goal of providing real-world evidence to guide the selection of antihypertensive drugs.

## 2 Methods

### 2.1 Data source

FAERS is a spontaneous AE database, created to support the FDA’s post-marketing safety surveillance system for all approved drugs and therapeutic biological products. FAERS data are publicly available, and include demographic and administrative information, drug information, AEs, report sources, and patient outcomes (FDA Adverse Event Reporting System (FAERS): Latest Quarterly Data Files, 2022a). We used the online tool OpenVigil 2.1 (https://openvigil.sourceforge.net/) to query the FAERS database. OpenVigil 2.1 is a pharmacovigilance tool developed for data extraction, cleaning, mining and analysis on the FAERS database (Bohm et al., 2012; Böhm and Herdegen, 2016). Besides, it now contains FAERS data from Q1/2004-Q3/2021.

### 2.2 Identifying antihypertensive drugs

OpenVigil 2.1 has already mapped arbitrary drug names (brand names, generic names, abbreviations, and so on) to a unique drugname by using Drugs@FDA and Drugbank. Thus, we firstly identified the drugname of each antihypertensive drug in the “Browse Window” of OpenVigil 2.1. Then we searched the drugname of each antihypertensive drug in the “OpenVigil Search Window”, respectively. The classification and detailed drugnames of antihypertensive drugs could be seen in Supplementary Table S1.

### 2.3 Definition of hyperuricaemia, gout and related AEs

In the FAERS database, AEs are coded according to Preferred Terms (PTs) derived from the Medical Dictionary for Regulatory Activities (MedDRA) terminology. In our study, hyperuricaemia, gout and related AEs were defined by 5 PTs: hyperuricaemia, gout, gouty arthritis, gouty tophus, and urate nephropathy (MedDRA. Introductory Guide MedDRA Version 22.1, 2022).

### 2.4 Statistical analysis

Disproportionality analysis was conducted by using OpenVigil 2.1. In the “Data Presentation And Statistics Box” of OpenVigil 2.1, proportional reporting ratio (PRR) was calculated to assess the association between individual antihypertensive drugs and the occurrences of hyperuricaemia, gout and related AEs. A higher PRR indicates a stronger association, and PRR = 2 suggests that the AE is two times more frequent in the users of target drug than in the background population. According to the criteria of Evans et al. (2001), a positive signal of disproportionality was defined as PRR of two or greater, chi-squared of at least four, and three or more cases (Evans et al., 2001).

## 3 Results

### 3.1 Overview of AE reports submitted for hyperuricaemia, gout and related AEs

The overview of AE reports submitted for hyperuricaemia, gout and related AEs in FAERS is shown in Table 1. The numbers of AE reports for hyperuricaemia, gout, gouty arthritis, gouty tophus and urate nephropathy were 1727, 8159, 446, 109, and 53 respectively. More males than females reported the above AEs except urate nephropathy. The majority of these AE reports were distributed in the 41–65 years and >65 years age groups. The main reporter country was the United States for these AEs except hyperuricaemia. After disproportionality analyses, the numbers of antihypertensive drugs with positive signals for hyperuricaemia, gout, gouty arthritis, gouty tophus and urate nephropathy were 46, 66, 27, 8, and 6, respectively.

**TABLE 1:** Overview of AE reports submitted for hyperuricaemia, gout and related AEs in FAERS.

	Hyperuricaemia	Gout	Gouty arthritis	Gouty tophus	Urate nephropathy
**Number of AE reports (n)**	1727	8159	446	109	53
Gender (n, %)					
Male	960 (55.59%)	4,673 (57.27%)	251 (56.28%)	61 (55.96%)	19 (35.85%)
Female	541 (31.33%)	2,873 (35.21%)	158 (35.43%)	44 (40.37%)	25 (47.17%)
Unknown	226 (13.09%)	613 (7.51%)	37 (8.30%)	4 (3.67%)	9 (16.98%)
Age (n, %)					
0–17 years	70 (4.05%)	8 (0.10%)	0 (0.00%)	6 (5.50%)	1 (1.89%)
18–40 years	128 (7.41%)	250 (3.06%)	17 (3.81%)	14 (12.84%)	3 (5.66%)
41–65 years	626 (36.25%)	2,311 (28.32%)	181 (40.58%)	28 (25.69%)	24 (45.28%)
>65 years	485 (28.08%)	2,301 (28.20%)	135 (30.27%)	37 (33.94%)	7 (13.21%)
Unknown	418 (24.20%)	3,289 (40.31%)	113 (25.34%)	24 (22.02%)	18 (33.96%)
Reporter country (n, %)					
United States	371 (21.48%)	5,600 (68.64%)	247 (55.38%)	56 (51.38%)	36 (67.92%)
Other countries	1,262 (73.07%)	2,285 (28.01%)	156 (34.98%)	46 (42.20%)	14 (26.42%)
Unknown	94 (5.44%)	394 (4.83%)	43 (9.64%)	7 (6.42%)	3 (5.66%)
**Number of antihypertensive drugs with positive signals (n)**	47	68	27	8	6

AE, adverse event; FAERS, FDA, adverse event reporting system.

### 3.2 Positive signals for hyperuricaemia

For diuretics, statistically significant signals emerged in 13 drugs: azosemide, furosemide, torasemide, bumetanide, hydrochlorothiazide, trichlormethiazide, indapamide, metolazone, xipamide, spironolactone, amiloride, triamterene, and eplerenone. For α blockers, statistically significant signals emerged in one drug: terazosin. For β blockers, statistically significant signals emerged in five drugs: acebutolol, atenolol, bisoprolol, metoprolol, and nebivolol. For α and β blockers, statistically significant signals emerged in one drug: carvedilol. For calcium channel blockers, statistically significant signals emerged in nine drugs: amlodipine, cilnidipine, azelnidipine, benidipine, lercanidipine, nifedipine, nilvadipine, nitrendipine, and diltiazem. For angiotensin converting enzyme inhibitors, statistically significant signals emerged in five drugs: captopril, enalapril, imidapril, temocapril, and trandolapril. For angiotensin II receptor blockers, statistically significant signals emerged in seven drugs: azilsartan medoxomil, candesartan, irbesartan, losartan, olmesartan, telmisartan, and valsartan. For renin inhibitors, statistically significant signals emerged in one drug: aliskiren. For compound preparations, statistically significant signals emerged in four drugs: Diovan HCT (valsartan and hydrochlorothiazide), Hyzaar (losartan potassium and hydrochlorothiazide), Exforge (amlodipine and valsartan), and Exforge HCT (amlodipine, valsartan and hydrochlorothiazide) (Table 2).

**TABLE 2:** Antihypertensive drugs with positive signals for hyperuricaemia.

Drug class	Drug name	N	PRR	χ^2^
Diuretics	Azosemide	5	25.35	93.60
	Furosemide	121	5.45	404.53
	Torasemide	29	10.85	245.41
	Bumetanide	5	3.77	7.57
	Hydrochlorothiazide	90	4.13	199.53
	Trichlormethiazide	11	64.76	624.32
	Indapamide	11	7.25	52.88
	Metolazone	4	4.99	9.07
	Xipamide	4	36.65	105.11
	Spironolactone	54	7.07	266.52
	Amiloride	18	43.05	690.64
	Triamterene	9	4.07	17.76
	Eplerenone	13	17.11	180.04
α blockers	Terazosin	6	5.36	17.09
β blockers	Acebutolol	3	9.20	14.46
	Atenolol	30	2.86	33.76
	Bisoprolol	80	8.97	532.79
	Metoprolol	56	2.33	39.88
	Nebivolol	15	6.59	65.01
α and β blockers	Carvedilol	32	3.65	57.83
Calcium channel blockers	Amlodipine	213	7.88	1,115.10
	Cilnidipine	15	151.62	2078.43
	Azelnidipine	6	59.27	286.95
	Benidipine	8	74.35	505.60
	Lercanidipine	10	9.72	69.35
	Nifedipine	40	10.11	311.74
	Nilvadipine	3	137.76	281.58
	Nitrendipine	4	30.00	84.81
	Diltiazem	15	2.36	10.34
Angiotensin converting enzyme inhibitors	Captopril	4	4.74	8.33
	Enalapril	34	7.00	165.54
	Imidapril	3	52.00	103.23
	Temocapril	3	120.65	246.00
	Trandolapril	4	9.28	21.79
Angiotensin II receptor blockers	Azilsartan medoxomil	12	32.56	333.96
	Candesartan	42	10.08	326.33
	Irbesartan	36	9.08	245.47
	Losartan	44	3.52	74.94
	Olmesartan	50	8.97	336.24
	Telmisartan	40	14.20	466.55
	Valsartan	81	3.78	155.08
Renin inhibitors	Aliskiren	9	8.01	48.16
Compound preparations	Diovan HCT (valsartan and hydrochlorothiazide)	5	3.88	7.97
	Hyzaar (losartan potassium and hydrochlorothiazide)	4	4.36	7.26
	Exforge (amlodipine and valsartan)	5	5.17	12.88
	Exforge HCT (amlodipine, valsartan and hydrochlorothiazide)	5	16.99	59.93

PRR, proportional reporting ratio; χ^2^, chi-square.

### 3.3 Positive signals for gout

For diuretics, statistically significant signals emerged in 11 drugs: furosemide, torasemide, bumetanide, hydrochlorothiazide, bendroflumethiazide, indapamide, metolazone, chlorthalidone, spironolactone, triamterene, and eplerenone. For antihypertensive drugs with central action, statistically significant signals emerged in one drug: clonidine. For α blockers, statistically significant signals emerged in two drugs: doxazosin and terazosin. For β blockers, statistically significant signals emerged in six drugs: acebutolol, atenolol, bisoprolol, metoprolol, nebivolol, and sotalol. For α and β blockers, statistically significant signals emerged in two drugs: carvedilol and labetalol. For calcium channel blockers, statistically significant signals emerged in six drugs: amlodipine, felodipine, lercanidipine, nifedipine, verapamil, and diltiazem. For angiotensin converting enzyme inhibitors, statistically significant signals emerged in 10 drugs: accupril, benazepril, captopril, enalapril, fosinopril, lisinopril, perindopril, quinapril, ramipril, and trandolapril. For angiotensin II receptor blockers, statistically significant signals emerged in eight drugs: azilsartan medoxomil, candesartan, eprosartan, irbesartan, losartan, olmesartan, telmisartan, and valsartan. For renin inhibitors, statistically significant signals emerged in one drug: aliskiren. For vasodilators, statistically significant signals emerged in two drugs: hydralazine and nitroglycerin. For compound preparations, statistically significant signals emerged in 15 drugs: Atacand HCT (candesartan and hydrochlorothiazide), Diovan HCT (valsartan and hydrochlorothiazide), Hyzaar (losartan potassium and hydrochlorothiazide), Micardis HCT (telmisartan and hydrochlorothiazide), Micardis plus (telmisartan and amlodipine), Tekturna HCT (aliskiren and hydrochlorothiazide), Azor (amlodipine and olmesartan), Exforge (amlodipine and valsartan), Exforge HCT (amlodipine, valsartan and hydrochlorothiazide), Entresto (sacubitril and valsartan), Lotrel (amlodipine and benazepril), Tenoretic 100 (atenolol and chlorthalidone), Caduet (amlodipine and atorvastatin), Edarbyclor (azilsartan medoxomil and chlorthalidone), Bidil (isosorbide dinitrate and hydralazine) (Table 3).

**TABLE 3:** Antihypertensive drugs with positive signals for gout.

Drug class	Drug name	N	PRR	χ^2^
Diuretics	Furosemide	611	5.85	2,271.93
	Torasemide	77	6.05	316.97
	Bumetanide	56	8.97	386.44
	Hydrochlorothiazide	398	3.85	797.91
	Bendroflumethiazide	15	2.46	11.62
	Indapamide	39	5.44	136.18
	Metolazone	55	14.59	678.68
	Chlorthalidone	22	5.00	66.35
	Spironolactone	128	3.49	221.37
	Triamterene	49	4.69	137.81
	Eplerenone	26	7.21	132.63
Antihypertensive drugs with central action	Clonidine	59	2.92	72.20
α blockers	Doxazosin	47	3.61	85.65
	Terazosin	17	3.21	23.68
β blockers	Acebutolol	6	3.89	10.15
	Atenolol	167	3.38	271.46
	Bisoprolol	194	4.50	512.20
	Metoprolol	446	4.02	955.07
	Nebivolol	40	3.70	75.91
	Sotalol	13	2.01	5.59
α and β blockers	Carvedilol	170	4.11	389.50
	Labetalol	14	2.80	14.48
Calcium channel blockers	Amlodipine	504	3.69	923.75
	Felodipine	20	4.23	46.10
	Lercanidipine	22	4.51	56.57
	Nifedipine	57	3.00	73.46
	Verapamil	48	2.77	52.25
	Diltiazem	95	3.17	137.71
Angiotensin converting enzyme inhibitors	Accupril	25	8.70	162.30
	Benazepril	46	3.79	91.38
	Captopril	10	2.50	7.59
	Enalapril	71	3.06	95.58
	Fosinopril	16	5.66	56.67
	Lisinopril	370	3.46	614.45
	Perindopril	36	2.77	38.86
	Quinapril	40	5.97	159.73
	Ramipril	205	4.32	507.60
	Trandolapril	6	2.94	5.87
Angiotensin II receptor blockers	Azilsartan medoxomil	13	7.43	65.96
	Candesartan	81	4.05	181.64
	Eprosartan	7	14.19	73.12
	Irbesartan	58	3.05	77.49
	Losartan	179	3.02	234.84
	Olmesartan	90	3.36	145.02
	Telmisartan	41	3.02	53.30
	Valsartan	446	4.44	1,121.23
Renin inhibitors	Aliskiren	21	3.95	43.19
Vasodilators	Hydralazine	57	6.01	231.37
	Nitroglycerin	71	4.15	165.07
Compound preparations	Atacand HCT (candesartan and hydrochlorothiazide)	12	13.14	122.64
	Diovan HCT (valsartan and hydrochlorothiazide)	28	4.60	75.12
	Hyzaar (losartan potassium and hydrochlorothiazide)	12	2.77	11.82
	Micardis HCT (telmisartan and hydrochlorothiazide)	7	6.91	29.69
	Micardis plus (telmisartan and amlodipine)	3	13.32	22.98
	Tekturna HCT (aliskiren and hydrochlorothiazide)	3	15.93	28.38
	Azor (amlodipine and olmesartan)	6	4.99	15.37
	Exforge (amlodipine and valsartan)	11	2.41	7.68
	Exforge HCT (amlodipine, valsartan and hydrochlorothiazide)	6	4.31	12.10
	Entresto (sacubitril and valsartan)	211	4.60	576.65
	Lotrel (amlodipine and benazepril)	23	5.28	75.35
	Tenoretic 100 (atenolol and chlorthalidone)	6	21.27	96.53
	Caduet (amlodipine and atorvastatin)	9	4.23	19.06
	Edarbyclor (azilsartan medoxomil and chlorthalidone)	10	19.75	159.71
	Bidil (isosorbide dinitrate and hydralazine)	3	21.68	40.32

PRR, proportional reporting ratio; χ^2^, chi-square.

### 3.4 Positive signals for gouty arthritis

For diuretics, statistically significant signals emerged in eight drugs: furosemide, torasemide, hydrochlorothiazide, indapamide, metolazone, spironolactone, triamterene, and eplerenone. For α blockers, statistically significant signals emerged in one drug: terazosin. For β blockers, statistically significant signals emerged in three drugs: bisoprolol, metoprolol, and nebivolol. For α and β blockers, statistically significant signals emerged in one drug: carvedilol. For calcium channel blockers, statistically significant signals emerged in two drugs: amlodipine and diltiazem. For angiotensin converting enzyme inhibitors, statistically significant signals emerged in six drugs: benazepril, captopril, enalapril, lisinopril, perindopril, and ramipril. For angiotensin II receptor blockers, statistically significant signals emerged in four drugs: candesartan, losartan, telmisartan, and valsartan. For vasodilators, statistically significant signals emerged in one drug: nitroglycerin. For compound preparations, statistically significant signals emerged in one drug: Entresto (sacubitril and valsartan) (Table 4).

**TABLE 4:** Antihypertensive drugs with positive signals for gouty arthritis.

Drug class	Drug name	N	PRR	χ^2^
Diuretics	Furosemide	48	8.72	285.87
	Torasemide	10	14.57	110.64
	Hydrochlorothiazide	42	7.81	219.80
	Indapamide	7	18.04	94.77
	Metolazone	9	44.28	331.82
	Spironolactone	30	15.78	373.77
	Triamterene	7	12.38	61.27
	Eplerenone	4	20.41	55.21
α blockers	Terazosin	4	13.92	35.57
β blockers	Bisoprolol	6	2.52	4.02
	Metoprolol	30	5.02	86.21
	Nebivolol	8	13.73	80.61
α and β blockers	Carvedilol	27	12.46	256.57
Calcium channel blockers	Amlodipine	27	3.61	45.35
	Diltiazem	15	9.38	100.48
Angiotensin converting enzyme inhibitors	Benazepril	10	15.34	117.41
	Captopril	8	37.27	242.80
	Enalapril	10	7.99	53.15
	Lisinopril	35	6.20	135.70
	Perindopril	6	8.53	32.24
	Ramipril	17	6.65	73.08
Angiotensin II receptor blockers	Candesartan	9	8.33	49.89
	Losartan	12	3.72	20.67
	Telmisartan	7	9.55	44.63
	Valsartan	29	5.34	91.57
Vasodilators	Nitroglycerin	18	19.87	291.61
Compound preparations	Entresto (sacubitril and valsartan)	10	3.98	18.96

PRR, proportional reporting ratio; χ^2^, chi-square.

### 3.5 Positive signals for gouty tophus

For diuretics, statistically significant signals emerged in three drugs: furosemide, torasemide, and spironolactone. For α blockers, statistically significant signals emerged in one drug: doxazosin. For calcium channel blockers, statistically significant signals emerged in two drugs: amlodipine and diltiazem. For angiotensin converting enzyme inhibitors, statistically significant signals emerged in one drug: enalapril. For vasodilators, statistically significant signals emerged in one drug: nitroglycerin (Table 5).

**TABLE 5:** Antihypertensive drugs with positive signals for gouty tophus.

Drug class	Drug name	N	PRR	χ^2^
Diuretics	Furosemide	25	21.52	361.13
	Torasemide	3	17.98	31.71
	Spironolactone	7	15.02	73.06
α blockers	Doxazosin	3	17.63	31.00
Calcium channel blockers	Amlodipine	6	3.26	6.85
	Diltiazem	9	24.24	163.22
Angiotensin converting enzyme inhibitors	Enalapril	8	27.60	166.14
Vasodilators	Nitroglycerin	4	18.00	46.53

PRR, proportional reporting ratio; χ^2^, chi-square.

### 3.6 Positive signals for urate nephropathy

For diuretics, statistically significant signals emerged in two drugs: furosemide and hydrochlorothiazide. For β blockers, statistically significant signals emerged in one drug: metoprolol. For calcium channel blockers, statistically significant signals emerged in one drug: amlodipine. For angiotensin II receptor blockers, statistically significant signals emerged in one drug: olmesartan. For compound preparations, statistically significant signals emerged in one drug: Benicar HCT (olmesartan and hydrochlorothiazide) (Table 6).

**TABLE 6:** Antihypertensive drugs with positive signals for urate nephropathy.

Drug class	Drug name	N	PRR	χ^2^
Diuretics	Furosemide	6	9.23	32.00
	Hydrochlorothiazide	3	4.51	4.74
β blockers	Metoprolol	4	5.68	10.20
Calcium channel blockers	Amlodipine	5	5.83	13.95
Angiotensin II receptor blockers	Olmesartan	3	18.06	30.88
Compound preparations	Benicar HCT (olmesartan and hydrochlorothiazide)	3	96.90	185.71

PRR, proportional reporting ratio; χ^2^, chi-square.

### 3.7 Drugs with positive signal for more than one AEs

All these five AEs were detected as positive signal in two drugs: furosemide and amlodipine. Four of these AEs were detected as positive signal in six drugs: torasemide, hydrochlorothiazide, spironolactone, metoprolol, diltiazem, and enalapril. Three of these AEs were detected as positive signal in 15 drugs: indapamide, metolazone, triamterene, eplerenone, terazosin, bisoprolol, nebivolol, carvedilol, captopril, candesartan, losartan, olmesartan, telmisartan, valsartan, and nitroglycerin. Two of these AEs were detected as positive signal in 19 drugs: bumetanide, doxazosin, acebutolol, atenolol, lercanidipine, nifedipine, benazepril, lisinopril, perindopril, ramipril, trandolapril, azilsartan medoxomil, irbesartan, aliskiren, Diovan HCT (valsartan and hydrochlorothiazide), Hyzaar (losartan potassium and hydrochlorothiazide), Exforge (amlodipine and valsartan), Exforge HCT (amlodipine, valsartan and hydrochlorothiazide), and Entresto (sacubitril and valsartan). In total, 42 drugs had positive signal for more than one AEs (Table 7).

**TABLE 7:** Antihypertensive drugs with positive signals for more than one AEs.

Drug class	Drug name	Hyperuricaemia	Gout	Gouty arthritis	Gouty tophus	Urate nephropathy
		PRR	PRR	PRR	PRR	PRR
Diuretics	Furosemide	5.45	5.85	8.72	21.52	9.23
	Torasemide	10.85	6.05	14.57	17.98	—
	Hydrochlorothiazide	4.13	3.85	7.81	—	4.51
	Spironolactone	7.07	3.49	15.78	15.02	—
	Indapamide	7.25	5.44	18.04	—	—
	Metolazone	4.99	14.59	44.28	—	—
	Triamterene	4.07	4.69	12.38	—	—
	Eplerenone	17.11	7.21	20.41	—	—
	Bumetanide	3.77	8.97	—	—	—
α blockers	Terazosin	5.36	3.21	13.92	—	—
	Doxazosin	—	3.61	—	17.63	—
β blockers	Metoprolol	2.33	4.02	5.02	—	5.68
	Bisoprolol	8.97	4.50	2.52	—	—
	Nebivolol	6.59	3.70	13.73	—	—
	Acebutolol	9.20	3.89	—	—	—
	Atenolol	2.86	3.38	—	—	—
α and β blockers	Carvedilol	3.65	4.11	12.46	—	—
Calcium channel blockers	Amlodipine	7.88	3.69	3.61	3.26	5.83
	Diltiazem	2.36	3.17	9.38	24.24	—
	Lercanidipine	9.72	4.51	—	—	—
	Nifedipine	10.11	3.00	—	—	—
Angiotensin converting enzyme inhibitors	Enalapril	7.00	3.06	7.99	27.60	—
	Captopril	4.74	2.50	37.27	—	—
	Benazepril	—	3.79	15.34	—	—
	Lisinopril	—	3.46	6.20	—	—
	Perindopril	—	2.77	8.53	—	—
	Ramipril	—	4.32	6.65	—	—
	Trandolapril	9.28	2.94	—	—	—
Angiotensin II receptor blockers	Candesartan	10.08	4.05	8.33	—	—
	Losartan	3.52	3.02	3.72	—	—
	Olmesartan	8.97	3.36	—	—	18.06
	Telmisartan	14.20	3.02	9.55	—	—
	Valsartan	3.78	4.44	5.34	—	—
	Azilsartan medoxomil	32.56	7.43	—	—	—
	Irbesartan	9.08	3.05	—	—	—
Renin inhibitors	Aliskiren	8.01	3.95	—	—	—
Vasodilators	Nitroglycerin	—	4.15	19.87	18.00	—
Compound preparations	Diovan HCT (valsartan and hydrochlorothiazide)	3.88	4.60	—	—	—
	Hyzaar (losartan potassium and hydrochlorothiazide)	4.36	2.77	—	—	—
	Exforge (amlodipine and valsartan)	5.17	2.41	—	—	—
	Exforge HCT (amlodipine, valsartan and hydrochlorothiazide)	16.99	4.31	—	—	—
	Entresto (sacubitril and valsartan)	—	4.60	3.98	—	—

AEs, adverse events; PRR, proportional reporting ratio; — indicates negative signals.

## 4 Discussion

### 4.1 Main findings

To our knowledge, this study provides the most comprehensive assessment of the association between antihypertensive drugs and the occurrences of hyperuricaemia, gout and related AEs. In our study, the numbers of antihypertensive drugs with positive signals for hyperuricaemia, gout, gouty arthritis, gouty tophus and urate nephropathy were 46, 66, 27, 8, and 6, respectively. These drugs included diuretics, antihypertensive drugs with central action, α blockers, β blockers, α and β blockers, calcium channel blockers, angiotensin converting enzyme inhibitors, angiotensin II receptor blockers, renin inhibitors, vasodilators, and compound preparations. Moreover, 42 drugs had positive signal for more than one AEs.

### 4.2 Explain the findings

The association of hyperuricaemia and gout with all classes of diuretics except potassium-sparing diuretics has been noted for decades. For example, a population-based cohort study in the United States suggested that uses of loop diuretics and thiazide diuretics were associated with increased risk of hyperuricaemia and gout (McAdams DeMarco et al., 2012). Moreover, a population-based case-control study of data from the UK General Practice Research Database showed that uses of loop diuretics, thiazide diuretics and thiazide-like diuretics were associated with increased risk of gout, whereas use of potassium-sparing diuretics was not (Bruderer et al., 2014). However, in our study, we not only found loop diuretics (azosemide, furosemide, torasemide and bumetanide), thiazide diuretics (bendroflumethiazide, hydrochlorothiazide and trichlormethiazide), and thiazide-like diuretics (chlorthalidone, indapamide, metolazone and xipamide) were associated with increased risk of hyperuricaemia, gout or related AEs, but also found some potassium-sparing diuretics (amiloride, spironolactone, triamterene and eplerenone) were associated with increased risk of hyperuricaemia, gout or related AEs. As the sample size of potassium-sparing diuretic users in previous studies was relatively small and may not have enough statistical power to determine the real association, further large sample studies are still needed to verify the association between potassium-sparing diuretics and the occurrences of hyperuricaemia, gout and related AEs. The mechanisms by which diuretics increase the risk of hyperuricaemia, gout and related AEs include the following points: first, diuretics produce salt and water loss to lead to volume depletion, which augments uric acid reabsorption; (Ben Salem et al., 2017) second, diuretics can affect ion exchanger proteins at the proximal tubule lumen membrane in the kidney, which would increase uric acid reabsorption and thus increase serum uric acid levels (McAdams DeMarco et al., 2012).

Our study also found that a series of α blockers, β blockers, angiotensin converting enzyme inhibitors and non-losartan angiotensin II receptor blockers were associated with increased risk of hyperuricaemia, gout or related AEs. These findings are consistent with most previous studies. For example, the previously mentioned cross-sectional study in Japan showed that uses of α blockers and β blockers were associated with increased risk of hyperuricaemia (Ueno et al., 2016). Moreover, the previously mentioned nested case-control study of data from the UK general practice database showed that β blockers, angiotensin converting enzyme inhibitors and non-losartan angiotensin II receptor blockers increased the risk of gout (Choi et al., 2012). Thus, the potential risks of hyperuricaemia, gout and related AEs associated with these antihypertensive drugs should be given adequate attention and close monitoring in future clinical practice. Several studies have already investigated the underling mechanisms by which these drugs elevate serum uric acid levels. For example, α blockers might raise angiotensin II levels, which would elevate serum uric acid levels *via* enhanced reabsorption of uric acid reabsorption (Ueno et al., 2016). In addition, β blockers can induce vasoconstriction in the nephrons, which would reduce glomerular filtration rate and thus elevate serum uric acid levels (Ueno et al., 2016).

In our study, the most startling findings were that both losartan and some calcium channel blockers were associated with increased risk of hyperuricaemia, gout or related AEs. These findings are inconsistent with most previous studies. Several epidemiological studies suggested that losartan, amlodipine, nifedipine, felodipine, nitrendipine, verapamil, and diltiazem could decrease serum uric acid levels (Daskalopoulou et al., 2005; Liu et al., 2021). Moreover, the previously mentioned nested case-control study of data from the UK general practice database showed that both losartan and calcium channel blockers reduced the risk of gout (Choi et al., 2012). In addition, some experimental studies also indicated that losartan could promoting uric acid excretion by inhibiting the function of urate/anion exchanger in the brush border of the renal proximal tubules, and calcium channel blockers could promoting uric acid excretion by increasing glomerular filtration rate (Daskalopoulou et al., 2005; Choi et al., 2012). Our findings changed the previous understanding about the effects of calcium channel blockers and losartan on hyperuricaemia and gout, and should be verified in future prospective studies.

Our study found for the first time that some antihypertensive drugs with central action (clonidine), α and β blockers (carvedilol and labetalol), renin inhibitors (aliskiren), and vasodilators (hydralazine and nitroglycerin) were associated with increased risk of hyperuricaemia, gout or related AEs. None of these drugs is commonly used as first-line antihypertensive drugs. Thus, the relatively small number of patients using these drugs may be a possible reason why previous studies have not noted the potential risks of hyperuricaemia, gout and related AEs associated with these drugs. Moreover, the mechanisms by which these drugs increase the risk of hyperuricaemia, gout and related AEs are unknown at present. Further studies are needed to confirm our findings and explore the underlying mechanisms.

Other findings of concern were that two drugs have positive signal for all these five pre-defined AEs and six drugs have positive signal for four of these AEs. Interestingly, most of these drugs have already been reported as risk factors for hyperuricaemia or gout in previous studies, which confirms the accuracy of our results. Besides, in our study, 15 drugs have positive signal for three of these AEs and 20 drugs have positive signal for two of these AEs. Although some of these drugs have not been noted in previous studies, the association between these drugs and the occurrence of hyperuricaemia, gout or related AEs is highly suspected and should attract enough attention in future clinical practice.

### 4.3 Clinical implications

Due to the limited and conflicting evidence, current guidelines only emphasize avoiding diuretics and using losartan or calcium channel blockers for treatment of hypertension in patients with hyperuricaemia or gout (Richette et al., 2016; Huang et al., 2020; Unger et al., 2020). However, our study suggested that both losartan and some calcium channel blockers were associated with increased risks of hyperuricaemia, gout and gouty arthritis. Thus, according to our results, the recommendation of using losartan or calcium channel blockers for treatment of hypertension in patients with hyperuricaemia or gout may not be appropriate.

### 4.4 Strengths and limitations

Our study has several strengths. First, FAERS includes all AE reports submitted to FDA, and the sample size is sufficient to identify rare adverse reactions. Second, our study detected signals for all classes of antihypertensive drugs (including both commonly used and less commonly used antihypertensive drugs), which could enable a comprehensive assessment of the association between various antihypertensive drugs and the occurrences of hyperuricaemia, gout and related AEs. Third, our study provided novel insight into the potential risks of hyperuricaemia, gout and related AEs associated with antihypertensive drugs, which does not support the recommendation of current guidelines.

Our study also has some limitations. First, we were unable to analyze dosage information in our study, as most AE reports failed to record this information. Second, the results of disproportionality analysis can only reveal associations but not causations. Thus, further epidemiological studies are still needed to confirm our findings. Third, our study failed to consider the potential confounding effect of hypertension on the association between antihypertensive drugs and the occurrences of hyperuricaemia, gout and related AEs, as hyperuricaemia is known to be associated to hypertension (Stewart et al., 2019). Future epidemiological studies should try to minimize the potential confounding effect of hypertension.

## 5 Conclusion

Our analysis of the FAERS database suggests that a series of diuretics, antihypertensive drugs with central action, α blockers, β blockers, α and β blockers, calcium channel blockers, angiotensin converting enzyme inhibitors, angiotensin II receptor blockers, renin inhibitors, vasodilators and compound preparations may be associated with increased risk of hyperuricaemia, gout or related AEs. Among these drugs, the positive associations for some potassium-sparing diuretics, calcium channel blockers and losartan are inconsistent with most previous studies, and the positive associations for some antihypertensive drugs with central action, α and β blockers, renin inhibitors and vasodilators are first reported in our study. These findings complement real-world evidence on the potential risks of hyperuricaemia, gout and related AEs associated with antihypertensive drugs.

## Data Availability

The original contributions presented in the study are included in the article/Supplementary Material, further inquiries can be directed to the corresponding authors.

## References

[B1] Ben Salem C. Slim R. Fathallah N. Hmouda H. (2017). Drug-induced hyperuricaemia and gout. Rheumatol. Oxf. 56, 679–688. 10.1093/rheumatology/kew293 27498351

[B2] Böhm R. Herdegen T. (2016). Using the OpenVigil 2 pharmacovigilance tool for guidance for clinical decisions involving newly occurring adverse events. GPTS Congr. Poster #408Accessed 2022 Available at: http://openvigil.sourceforge.net/doc/2016-poster-dgpt-openvigil-decision-making-6-no-pix.pdf .

[B3] Bohm R. Hocker J. Cascorbi I. Herdegen T. (2012). OpenVigil-free eyeballs on AERS pharmacovigilance data. Nat. Biotechnol. 30, 137–138. 10.1038/nbt.2113 22318027

[B4] Bruderer S. Bodmer M. Jick S. S. Meier C. R. (2014). Use of diuretics and risk of incident gout: A population-based case-control study. Arthritis Rheumatol. 66, 185–196. 10.1002/art.38203 24449584

[B5] Choi H. K. Soriano L. C. Zhang Y. Rodriguez L. A. (2012). Antihypertensive drugs and risk of incident gout among patients with hypertension: Population based case-control study. BMJ 344, d8190. 10.1136/bmj.d8190 22240117 PMC3257215

[B6] Cicero A. F. Salvi P. D'Addato S. Rosticci M. Borghi C. (2014). Association between serum uric acid, hypertension, vascular stiffness and subclinical atherosclerosis: Data from the brisighella heart study. J. Hypertens. 32, 57–64. 10.1097/HJH.0b013e328365b916 24309486

[B7] Daskalopoulou S. S. Tzovaras V. Mikhailidis D. P. Elisaf M. (2005). Effect on serum uric acid levels of drugs prescribed for indications other than treating hyperuricaemia. Curr. Pharm. Des. 11, 4161–4175. 10.2174/138161205774913309 16375738

[B8] Duggirala H. J. Tonning J. M. Smith E. Bright R. A. Baker J. D. Ball R. (2016). Use of data mining at the food and drug administration. J. Am. Med. Inf. Assoc. 23, 428–434. 10.1093/jamia/ocv063 PMC11740535 26209436

[B9] Evans S. J. Waller P. C. Davis S. (2001). Use of proportional reporting ratios (PRRs) for signal generation from spontaneous adverse drug reaction reports. Pharmacoepidemiol Drug Saf. 10, 483–486. 10.1002/pds.677 11828828

[B10] FDA Adverse Event Reporting System (FAERS) Quarterly Data Extract Files (2022a). Available at: https://fis.fda.gov/extensions/FPD-QDE-FAERS/FPD-QDE-FAERS.html.(Accessed 2022).

[B11] FDA Adverse Event Reporting System (FAERS): Latest Quarterly Data Files (2022b). Available at: https://www.fda.gov/drugs/questions-and-answers-fdas-adverse-event-reporting-system-faers/fda-adverse-event-reporting-system-faers-latest-quarterly-data-files.(Accessed 2022).

[B12] Feig D. I. (2014). Serum uric acid and the risk of hypertension and chronic kidney disease. Curr. Opin. Rheumatol. 26, 176–185. 10.1097/BOR.0000000000000033 24419747

[B13] Huang Y. F. Yang K. H. Chen S. H. Xie Y. Huang C. B. Qing Y. F. (2020). Practice guideline for patients with hyperuricemia/gout. Zhonghua Nei Ke Za Zhi 59, 519–527. 10.3760/cma.j.cn112138-20200505-00449 32594685

[B14] Jiao X. F. Li H. L. Jiao X. Y. Guo Y. C. Zhang C. Yang C. S. (2020). Ovary and uterus related adverse events associated with statin use: An analysis of the FDA adverse event reporting system. Sci. Rep. 10, 11955. 10.1038/s41598-020-68906-2 32686733 PMC7371681

[B15] Liu J. Chen L. Yuan H. Huang K. Li G. Sun N. (2021). Survey on uric acid in Chinese subjects with essential hypertension (SUCCESS): A nationwide cross-sectional study. Ann. Transl. Med. 9, 27. 10.21037/atm-20-3458 33553320 PMC7859747

[B16] McAdams DeMarco M. A. Maynard J. W. Baer A. N. Gelber A. C. Young J. H. Alonso A. (2012). Diuretic use, increased serum urate levels, and risk of incident gout in a population-based study of adults with hypertension: The atherosclerosis risk in communities cohort study. Arthritis Rheum. 64, 121–129. 10.1002/art.33315 22031222 PMC3253199

[B17] MedDRA. Introductory Guide MedDRA Version 22.1 (2022). Available at: https://www.meddra.org/sites/default/files/guidance/file/000354_intguide_22.1.pdf. (Accessed 2022).

[B18] Richette P. Doherty M. Pascual E. Barskova V. Becce F. Castaneda-Sanabria J. (2016). 2016 updated EULAR evidence-based recommendations for the management of gout. Ann. Rheum. Dis. 76, 29–42. 10.1136/annrheumdis-2016-209707 27457514

[B19] Stewart D. J. Langlois V. Noone D. (2019). Hyperuricemia and hypertension: Links and risks. Integr. Blood Press Control 12, 43–62. 10.2147/IBPC.S184685 31920373 PMC6935283

[B20] Ueno S. Hamada T. Taniguchi S. Ohtani N. Miyazaki S. Mizuta E. (2016). Effect of antihypertensive drugs on uric acid metabolism in patients with hypertension: Cross-sectional cohort study. Drug Res. (Stuttg). 66, 628–632. 10.1055/s-0042-113183 27643410

[B21] Unger T. Borghi C. Charchar F. Khan N. A. Poulter N. R. Prabhakaran D. (2020). 2020 international society of hypertension global hypertension practice guidelines. Hypertension 75, 1334–1357. 10.1161/HYPERTENSIONAHA.120.15026 32370572

[B22] Verdecchia P. Schillaci G. Reboldi G. Santeusanio F. Porcellati C. Brunetti P. (2000). Relation between serum uric acid and risk of cardiovascular disease in essential hypertension. The PIUMA study. Hypertension 36, 1072–1078. 10.1161/01.hyp.36.6.1072 11116127

[B23] Zhu Y. Pandya B. J. Choi H. K. (2012). Comorbidities of gout and hyperuricemia in the US general population: Nhanes 2007-2008. Am. J. Med. 125, 679–687. 10.1016/j.amjmed.2011.09.033 22626509

