# Supplementary material for: Hyperuricaemia, gout and related adverse events associated with antihypertensive drugs: A real-world analysis using the FDA adverse event reporting system
Source: Front Pharmacol. 2023 Jan 9;13:1045561. doi: 10.3389/fphar.2022.1045561 (PMC9868414; doi:10.3389/fphar.2022.1045561)
Supplement: Supplementary file 1 [file Table1.DOCX]

**Supplementary Table 1.** The classification and detailed drugnames of antihypertensive drugs.

| Classification | Drugnames |
| --- | --- |
| Diuretics (n=18) | Azosemide, Furosemide, Torasemide, Bumetanide, Hydrochlorothiazide, Bendroflumethiazide, Methyclothiazide, Trichlormethiazide, Polythiazide, Indapamide, Metolazone, Chlorthalidone, Xipamide, Spironolactone, Amiloride, Triamterene, Eplerenone, Etacrynic acid |
| Antihypertensive drugs with central action (n=3) | Clonidine, Methyldopa, Guanfacine |
| α blockers (n=4) | Doxazosin, Prazosin, Terazosin, Phentolamine |
| β blockers (n=12) | Acebutolol, Atenolol, Esmolol, Bisoprolol, Betaxolol, Metoprolol, Nadolol, Nebivolol, Propranolol, Sotalol, Ethotoin, Pindolol |
| α and β blockers (n=2) | Carvedilol, Labetalol |
| Calcium channel blockers (n=16) | Amlodipine, Cilnidipine, Azelnidipine, Benidipine, Clevidipine, Felodipine, Lercanidipine, Nifedipine, Nilvadipine, Nitrendipine, Nisoldipine, Nicardipine, Isradipine, Lacidipine, Verapamil, Diltiazem |
| Angiotensin converting enzyme inhibitors (n=15) | Accupril, Benazepril, Captopril, Enalapril, Enalaprilat, Fosinopril, Imidapril, Lisinopril, Moexipril, Perindopril, Quinapril, Ramipril, Temocapril, Trandolapril, Cilazapril, |
| Angiotensin II receptor blockers (n=8) | Azilsartan medoxomil, Candesartan, Eprosartan, Irbesartan, Losartan, Olmesartan, Telmisartan, Valsartan |
| Renin inhibitors (n=1) | Aliskiren |
| Vasodilators (n=4) | Hydralazine, Nitroglycerin, Fenoldopam, Nitroprusside |
| Compound preparations (n=35) | Atacand HCT (candesartan and hydrochlorothiazide), Diovan HCT (valsartan and hydrochlorothiazide), Hyzaar (losartan potassium and hydrochlorothiazide), Micardis HCT (telmisartan and hydrochlorothiazide), Micardis plus (telmisartan and amlodipine), Tekturna HCT (aliskiren and hydrochlorothiazide), Azor (amlodipine and olmesartan), Exforge (amlodipine and valsartan), Exforge HCT (amlodipine, valsartan and hydrochlorothiazide), Entresto (sacubitril and valsartan), Lotrel (amlodipine and benazepril), Tenoretic 100 (atenolol and chlorthalidone), Caduet (amlodipine and atorvastatin), Edarbyclor (azilsartan medoxomil and chlorthalidone), Bidil (isosorbide dinitrate and hydralazine), Benicar HCT (olmesartan and hydrochlorothiazide), Ziac (bisoprolol and hydrochlorothiazide), Dutoprol (hydrochlorothiazide and metoprolol succinate), Lopressor HCT (hydrochlorothiazide and metoprolol tartrate), Corzide (benzfluorothiazide and nadolol), Byvalson (nebilol and valsartan), Tarka (trandolapril and verapamil hydrochloride), Propranolol hydrochloride and hydrochlorothiazide, Consensi (amlodipine and celecoxib), Prestalia (amlodipine and perindopril), Twynsta (amlodipine and telmisartan), Lotensin HCT (benazepril and hydrochlorothiazide), Accuretic (quinazepril and hydrochlorothiazide), Prinzide (lisinopril and hydrochlorothiazide), Uniretic (hydrochlorothiazide and mosiapril hydrochloride), Vaseretic (enalapril and hydrochlorothiazide), Avalide (irbesartan and hydrochlorothiazide), Teveten HCT (iprosartan mesylate and hydrochlorothiazide), Maxzide (triamphenteridine and hydrochlorothiazide), tribenzor (amlodipine, hydrochlorothiazide and olmesartan) |
